# Impact of the COVID-19 pandemic on child and adolescent mental health services

**DOI:** 10.1192/bjo.2021.976

**Published:** 2021-08-05

**Authors:** Hannah Chu-Han Huang, Dennis Ougrin

**Affiliations:** CAMHS Specialist Registrar, South London and Maudsley NHS Foundation Trust, UK; Institute of Psychiatry, Child and Adolescent Psychiatry, King's College London, UK

**Keywords:** Anxiety disorders, attention-deficit hyperactivity disorders, bipolar affective disorders, depressive disorders, eating disorders NOS

## Abstract

The COVID-19 pandemic and government lockdown restrictions have had an impact on children and young people worldwide. In this editorial, we explore how and why referrals to UK children and adolescent mental health services (CAMHS) have changed during the pandemic and summarise the emerging data on the potential reasons behind this.

The COVID-19 pandemic has affected all of us in one way or the other. The National Health Service (NHS) has had to adapt faster than any of us could have imagined, with unprecedented changes to services provided and a reduction in face-to-face patient care.^1^ Alongside the physical health burden of the pandemic, there is the emerging and arguably, more burdensome, mental health epidemic that we are observing across the country and the world. Children and young people have been disproportionately adversely affected, as they have had to adapt to extraordinary changes to the world around them. Throughout the pandemic, data has been emerging about how COVID-19 and government lockdown measures have had an impact on the mental health of the younger population and in turn, the impact on children and adolescent mental health services (CAMHS).

## How have referrals to CAMHS changed?

The paper in *BJPsych Open* by McNicholas et al,^2^ describes how government lockdown measures and restrictions during the COVID-19 pandemic in 2020 affected referral rates to CAMHS in the Republic of Ireland. The authors observed an initial drop in referrals in the months of March–May 2020 during the first lockdown period of 53% compared with the same period in 2019. Low attendance at general practitioners or emergency departments, and closure of schools have been cited as probable reasons for this. Similar referral patterns have been seen across the country in the UK in all psychiatric specialties and settings.^3^ An initial drop in presentations to paediatric emergency departments was also observed.^1^ A similar picture has also emerged from the USA in young people from predominantly Hispanic/Latinx backgrounds where reductions in symptoms of mental health problems were found in the first month after COVID-19 restrictions were implemented.^4^

However, McNicholas et al^2^ reported that from September 2020 onwards there was a sharp increase in referrals, with a peak of 180% in November 2020 compared with previous years. This has created ongoing demand on services already under strain that need to adapt their ways of working because of lockdown restrictions. With a pre-existing upwards trend in referrals to CAMHS before the COVID-19 pandemic,^5^ it is unclear how services will cope with this surge in demand.

## Why have referrals to CAMHS changed?

There are multiple reasons why children and young people are more vulnerable to the impact of the COVID-19 restrictions. School closures, increased social isolation, increased financial and emotional stress and greater exposure to family conflicts are some of the major risk factors of worsening mental health in this population.^5^ A study looking at primary school children found that financial strain within the family was linked to increased mental health difficulties in children because of an increase in parental anxiety and depression.^6^

McNicholas et al's paper^2^ described quantitively how referrals have changed; however, data regarding types of presentations, diagnoses and severity, are still emerging. We have summarised below some findings to date about how the presentation of certain psychiatric disorders have been affected as a result of the COVID-19 pandemic.

### Severity of presentations to CAMHS during COVID-19 pandemic

One study has found a higher in-patient admission rate for young people presenting to the emergency department with mental health problems.^7^ This could be because of a delay in presentation owing to lockdown restrictions meaning greater deterioration in mental state, or a lack of effective support from community CAMHS teams.

McNicholas et al^2^ also described how a higher proportion of referrals to their service were deemed urgent as well as increased numbers requiring onward referral to specialist CAMHS, including in-patient services. This would be in line with emerging evidence that young people are being referred with increasing complex and atypical presentations as a result of the pandemic as outlined below.

### Depression and anxiety

There is emerging evidence that levels of depression and anxiety have increased in children and adolescents as a result of COVID-19 lockdowns around the world.^8^ This is unsurprising as lockdown measures have meant greater social isolation, school closures and continuous levels of uncertainty imposed on the population. Similar studies in the UK have found increases in childhood depression but did not find any significant change in anxiety initially.^9^

On the contrary, it has been found that lockdown measures have led to a decrease in symptoms in some young people with social anxiety, providing them with a period of respite.^10^ However, they have subsequently experienced exacerbated symptoms, with the easing of lockdown restrictions and re-integration back to school and with peers. Particular attention to this population of young people who have thrived in lockdown may need to be taken in anticipation for their increased need for mental health services as normal life resumes post-pandemic. There may be a tendency for individuals and families to be hesitant to seek help for social anxiety symptoms as they may have had a lower level of impairment during the pandemic and the associated lockdown.^11^

### Self-harm and suicide

Despite advances in understanding and treatment,^12–14^ the prevalence of self-harm and suicide in young people had been on the increase before the pandemic struck. Although the National Child Mortality Database indicated a possible increase in child suicide during the first UK lockdown,^15^ self-harm presentations to emergency departments reduced very substantially at the beginning of the pandemic.^1^ The impact of the pandemic on suicide remains unclear, but early studies show no substantial increase.^16^ However, the longer-term impact of the pandemic and lockdown restrictions on this population remains unknown and we must remain vigilant in supporting these young people.

### Eating disorders

The COVID-19 pandemic has had a profound and negative impact on patients experiencing eating disorders especially anorexia nervosa.^17^ Risk factors identified include perceived lack of control, loss of routine, social isolation, food insecurities and pressures to exercise. As a result, there has been a significant increase in referrals and wait times for specialist services.^18^ Some clinicians have also observed increase need for admission and use of nasogastric tube feeding as a result of severe loss of body weight in a short period of time.

### Obsessive–compulsive disorder

A study by Nissen et al,^19^ showed a worsening of symptoms in adolescents with OCD associated with the worsening of anxiety, depressive symptoms and the extent of avoidance behaviour. Conversely, a study in Israel showed that there was no increase in severity of OCD symptoms in children and adolescents during the first COVID-19 lockdown.^20^ An explanation for this could be reduced exposure to contamination in the outside world because of lockdown measures or reduced stressors that would normally exacerbate symptoms.

### Tic disorder

Clinicians have seen an increase in presentations of sudden and new onset of severe tics and ‘tic-like’ attacks as reported by Heyman et al.^21^ Interestingly they report an atypical presentation of sudden onset of motor and phonic tics of a complex and bizarre nature in adolescent girls that are functional in nature and that may be related to the stress of the COVID-19 pandemic.

## How have services adapted to cope with changes and what further can be done?

The NHS and mental health services have had to adapt at unprecedented speed in order to cope with the COVID-19 pandemic. During the first lockdown, there were initiatives around the country to open specialist mental health emergency departments, including for children and adolescents, to keep the pressure off general hospitals. Although this was thought to be an efficient and safe idea at the time, the initial reduction in psychiatric presentations to the emergency department meant that this was not a cost-effective system, and many of these services have now been closed.

Mental health services, particularly community-based teams, have had to rely heavily on phone and video consultations, to provide services throughout the pandemic, with a reduction in face-to-face contact. Overall, there appears to have been a positive transition to the use of telepsychiatry,^22^ with it being viewed as a convenient and safe way to provide patient care. Telepsychiatry has been shown to be a useful tool in public health emergencies and it will be interesting to see if there continues to be a role for it after the pandemic.

With the surge in demand for CAMHS adding to an already strained service, it is clear that investment into the provision to sustain it is prudent. The UK Government announced in March 2021 an extra £79 million for CAMHS^23^ on top of the £1.4 billion pledged in 2015.^24^ However, this amount still represents a tiny proportion of total NHS spend. Much more needs to be done in order to mitigate the burden and impact that the pandemic has had on our young and most vulnerable people, in order to prevent longer-term implications in the future.
